# Assessment, pharmacological therapy and rehabilitation management of musculoskeletal pain in children with mucopolysaccharidoses: a scoping review

**DOI:** 10.1186/s13023-022-02402-w

**Published:** 2022-07-08

**Authors:** R. Gnasso, B. Corrado, I. Iommazzo, F. Migliore, G. Magliulo, B. Giardulli, C. Ruosi

**Affiliations:** Physical Medicine and Rehabilitation, Department of Public Health, University Federico II of Naples, Via S. Pansini, 5, 80131 Naples, Italy

**Keywords:** Mucopolysaccharidoses, Child, Pain, Musculoskeletal pain, Pharmacology, Rehabilitation

## Abstract

**Background:**

Pain of musculoskeletal origin is very common in young patients affected by Mucopolysaccharidoses. This scoping review evaluates the evidence for assessment, pharmacological treatment and rehabilitation management for musculoskeletal pain of the latter.

**Methods:**

A Medline search through PubMed has been performed for studies published in English at least for the past twenty years. Two investigators independently reviewed all search results and extracted those that met the inclusion criteria.

**Results:**

29 studies have been selected and analysed in depth, of which 10 related to pain assessment, 11 concerned pharmacological approach, and 8 reported rehabilitation approaches.

**Conclusion:**

Few data are available in literature concerning the classification and management of pain in children with Mucopolysaccharidoses. Notwithstanding, pain evaluation methods are effectively used to classify pain intensity, according to the age group and communication abilities of young Mucopolysaccharidoses patients. The review emphasizes that drug therapies have a palliative purpose, while rehabilitation reduces musculoskeletal pain and can provide a therapeutic effect on disabilities.

## Introduction

Mucopolysaccharidoses (MPSs) are a heterogeneous group of inborn hereditary progressive diseases caused by the absence or malfunctioning of enzymes involved in the physiological degradation of glycosaminoglycans (GAGs). Enzyme deficiency leads to lysosomal GAGs storage causing disabling musculoskeletal, visceral and neurological effects due to the exceeding activation of inflammatory agents’ cascade such as cytokines, chemokines, cathepsins S and Z, lysozyme M, CD38, DAP12, cytochrome b558, polineuronal microglial and complements C1q and C4. All these inflammatory agents then determine an extreme macrophage/monocyte response [1]. MPSs are currently distinguished based on the deficient enzyme. Their incidence is about 1 in 25,000 [2].

The presentation spectrum is highly different, from more severe to milder forms. Chronic pain is an experience that connects them all, above all due to bone and articular involvement, where the intracellular inflammation alters collagenase activity of cathepsin K, leading to synovial hyperplasia, cartilage apoptosis, modified connective tissue matrices, reduction of chondrocytes development, osteoblast differentiation, mineralization and, in the end, inflammatory joint destruction [3, 4]. Subjects with neurological impairment, in fact, suffer by some typical clinical deformities due to the massive GAGs accumulation in musculoskeletal tissues as bone cortex and trabecular matrix: craniofacial dysmorphisms (i.e., coarse face), claw hands, cervical stenosis genu and coxa valga, thoracolumbar scoliosis, pectus carinatum, dwarfism, kyphotic deformity, muscle contractures and joint stiffness (except for MPS IV characterized by hyperlaxity) are typically painful [5–7]. Physical impairment increases progressively, resulting in a heightened intensity of pain, which becomes a contributing cause of musculoskeletal disability, mostly in patients with serious neurocognitive delay. Major features of MPSs are listed in tables section (Table 1) [8].

**Table 1:** MPSs features [3]

MPS	Type	Incidence	Musculoskeletal features	Other major features
MPS I	Hurler	1:100,000	Disproportional short stature, joint stiffness/contractures,claw hands, odontoid hypoplasia, thoracolumbar kyphosis, scoliosis, hip dysplasia, genu valgum, Carpal tunnel syndrome, trigger fingers, Dysostosis multiplex	Psychomotor retardation, coarse facial features, macrocephaly, spinal cord compression, corneal clouding (vision impairment), hearing loss, organomegaly, cardiac (valve, coronary artery) disease, respiratory disease, recurrent ENT infections, umbilical/inguinal hernias, hydrocephalus
	Hurler-Scheie		Intermediate between MPS I Hurler and MPS I Scheie	Intermediate between MPS I Hurler and MPS I Scheie
	Scheie		Mild short stature, joint stiffness/contractures, carpal tunnel syndrome, trigger fingers, Dysostosis multiplex	Corneal clouding, cardiac (valve) disease, umbilical/inguinal hernias, organomegaly, spinal cord compression, hearing loss, No psychomotor retardation, Only mild coarsening of facial features
MPS II	Hunter A severe	1:100,000–150,000 (male subjects)	Disproportional short stature, joint stiffness/contractures Thoracolumbar kyphosis, hip dysplasia, Carpal tunnel syndrome, trigger fingers Dysostosis multiplex	Psychomotor retardation, coarse facial features, macrocephaly, respiratory disease, cardiac disease, retinal degeneration (no corneal clouding), hearing loss, organomegaly, Gastrointestinal symptoms (diarrhoea), umbilical/inguinal hernia Hydrocephalus, spinal cord compression, Melanocytosis
	Hunter B mild		Mild disproportional short stature, joint stiffness/ contractures, Carpal tunnel syndrome, Dysostosis multiplex	Hearing and vision impairment, Gastrointestinal symptoms (diarrhoea), sleep apnoea, No psychomotor retardation
MPS III	Sanfilippo A – D	1:70,000	Short stature, mild joint stiffness/contractures, Genu valgum, Dysostosis multiplex	Severe psychomotor deterioration and behaviour problems: progressive dementia, aggression, hyperactivity, sleeping disorders, Seizures, Mild somatic manifestations: coarse facial features, hirsutism, organomegaly, hearing loss
MPS IV	Morquio A–B	1:200,000	Disproportional short stature, hypermobile joints, Odontoid hypoplasia, thoracolumbar kyphosis, scoliosis, pectus carinatum, coxa valga, genu valgum, pes planus, Dysostosis multiplex	Hearing loss, corneal clouding, cardiac (valve) disease, organomegaly, caries teeth, spinal cord compression, No psychomotor retardation and no coarse facial features
MPS VI	Maroteaux–Lamy	1:250,000–600,000	Disproportional short stature, joint stiffness/contractures (mainly hips), Kyphoscoliosis, hip dysplasia, genu valgum, odontoid hypoplasia, carpal tunnel syndrome, trigger fingers, dysostosis multiplex	Corneal clouding, hearing loss, hernias, organomegaly, cardiomyopathy, cardiac valve disease, respiratory disease, Spinal cord compression, Coarse facial features, No psychomotor retardation
MPS VII	Sly	1:250,000	Disproportional short stature, joint stiffness/contractures, Odontoid hypoplasia, thoracolumbar kyphosis, dysostosis multiplex	Wide spectrum of severity: from severe hydrops fetalis to less severe phenotypes with (mild) psychomotor retardation, coarse facial features, corneal clouding, hernias, organomegaly, cardiac (valve) disease, spinal cord compression
MPS IX	Hyaluronidase deficiency	6 Case reported	Periarticular nodular soft tissue masses (extremities) with episodes of painful swelling, Short stature, no joint stifness	Mild facial changes (eg, flattened nasal bridge), No psychomotorretardation

Pain suffered by MPS patients is nociceptive (somatic or visceral), neuropathic and mixed [9]. Nociceptive pain is due to joint stiffness/swelling, main feature of all MPS (except for MPS IV and IX) and is related to GAGs accumulation in muscle, tendons, ligaments and epi/metaphyseal sites of bones [10]. Nociceptors, in addition, are continuously engaged by the chronic inflammation process that leads to neuropathic pain, as a consequence of the central/peripheral nervous system malfunction [11]. This stabbing pain is involved in carpal tunnel syndrome, the most common entrapment syndrome in MPS [12], in the tarsal tunnel syndrome and in the spinal cord compression with cervical stenosis due to GAGs storage in atlantoaxial joint, which is the most serious because it is associated to a high risk of subluxation, paraplegia and, above all, sudden death [13]. Rachis and brain involvement can be recorded by MRI showing, in some cases, reduction of spinal canal, frequently at C1-D7 and L5-S1 levels, anterior and posterior spondylolisthesis, herniated disks, hourglass deformation of brainstem and cortical atrophy in frontotemporal lobes [14].

Hallmark of MPS pain is Tumor Necrosis Factor alpha (TNF-α), involved in prostanoids release, like prostaglandin E2, with systemic disabling pain, reduced physical activity and fatigue [15].

Sanfilippo Syndrome is the MPS in which most commonly articular pain is observed (69%), above all hip pain (27.8%) and back pain (25.9%) [16]. In Morquio A syndrome, instead, a particular pain pattern has been showed: motor suffering was inversely related to the frequency of wheelchair use and the greater mobility was related to an earlier fatigue and joint pain [17].

To date, many Authors have underscored how much pain these patients experience, especially in paediatric MPS patients, however, assessment results are still underestimated and badly valued (Table 2) [12]. Therefore, there is a need to summarise current evidence about the pain assessment and management for children with MPS. The aim of this review is to: analyze the management of musculoskeletal pain in paediatric population affected by MPS within 18 years, investigate pain assessment tools in such population and evaluate options of both pharmacological and rehabilitative treatments.

**Table 2:** Data about pediatric population affected by MPS [4]

Authors	Patients	MPS	Musculoskeletal pain
Brans et al	89 adult and pediatric MPS subjects (55 of whom agreed to participate)	MPS I, MPS II, MPS III, MPS IV, MPS VI, MPS type unknown	69% of children reported joint pain, mainly hip (27,8%) and back pain (25,9%). The highest frequency of pain was observed in MPS III group (52.9%)
Hendriksz et al	Adult and pediatric MPS subjects with	Morquio A Syndrome (MPS IVA)	64% of children reported joint pain (spinal area (63%), lower extremities (100%), upper extremities (69%), and head and neck area (56%))
Vijay and Wraith	29 adult and pediatric MPS subjects	Attenuated MPSI phenotype	Progressive arthropathy (86%), fixed flexion deformity of the fingers (24%), and kyphosis, scoliosis, and/or lordosis (24%)
White and Sousa	18 pediatric MPS subjects	MPSIII	Many subjects requested orthopaedic evaluation of hip pain (hip dysplasia in 8 subjects; bilateral osteonecrosis of the femoral heads in 4 subjects)
de Ruijter et al	33 adult and pediatric MPS III subjects	MPS-3A, MPS-3B, MPS-3C	For 15 of the 33 subjects pain was indicated in one or both hips

## Method

### Study design

The scoping review protocol is based on: identifying the research question; searching relevant studies; selecting the studies; analysing the data; collating, summarizing and reporting the results.

### Eligibility criteria

This comprehensive review has included only articles published in English without exclusion for study design. Studies regarding pain assessments and management in MPS patients using appropriate instruments were included. Articles involving surgical approaches to MPS patients and the oldest or innovative therapies, which treat the disorder but have no impact on musculoskeletal pain control, were excluded. Studies concerning palliative treatments were included if the pharmacological approach assessment was based on the intensity of the perceived pain and if the rehabilitation intervention was defined as a physical, cognitive or social activity focused on improving the psychophysical condition of patients.

### Search strategy

Medline was searched for relevant studies on MPS pain evaluation and management published in English at least for the past 20 years. The sensitivity-maximising search strategy combined “key terms” such as mucopolysaccharidoses, children, pain, pain evaluation, scales, pain treatments, rehabilitation. When appropriate, the original source describing the analytical tools referenced in a study was verified.

### Data collection

Two independent authors assessed the titles and abstracts of the retrieved studies to screen out articles that did not meet the inclusion criteria. Any disagreement was resolved by discussion and the intervention of a third author.

Data reported are related, in the first part, to the evaluation scales divided by age groups and the level of young patients’ collaboration. The second part concerns palliative and rehabilitation treatments implemented so far and reported in the literature.

### Data analysis

The data concerning pain assessment methods, such as name scales or tests, were searched and reported from the included articles. Moreover, the data concerning pharmacological and rehabilitative approaches to treat this population were searched and reported. To examine the effects of these treatments, Mean Differences (MD), Standardized Main Difference (SMD) and Confidence Interval (CI) were estimated, when available. The grade of evidence was rated independently for each outcome by two authors using the Grading of Recommendations Assessment, Development and Evaluation (GRADE) system.

## Results

Following the assessment of eligibility, 29 articles were included in this review. There was good agreement between authors for the study selection: **t**wo authors selected the most pertinent articles, submitting them to each other and, in agreement, to a third author who objectively assessed the eligibility of the studies falling within the inclusion criteria. Three other authors supervised the final 29 articles resulting from the choices of the first three authors, approving and sharing them definitively.

Eight different pain assessment scales for children were reported in ten articles. Eleven articles concerned pain control with the use of a pharmacological approach. Eight articles, instead, assessed different rehabilitation approaches. At last, one article review regarded both drug therapies and rehabilitation techniques, focusing on how they all have a good impact on pain control and reducing the musculoskeletal disabilities of young patients and the perceived pain (Table 3).

**Table 3:** Therapeutic options in MPSs

Pharmacological management		
Source	Publication date	Drug admnistered
Clarke et al. [27]	2009	Laronidase (ERT)
Burton e al. [28]	2015	Elosulfase alpha (ERT)
Robinson et al. [29]	2002	Pamidronate (biphosphonate)
Polgreen et al. [30]	2017	Adalimumab (TNF alpha inibitor)
Congedi et al. [31]	2018	NSAIDs, Acetaminophen, Opioids
Politei et al. [32]	2016	ERT, NSAIDs, Acetaminophen, Opioids
Felleiter et al. [33]	2005	NSAIDs, Acetaminophen, Opioids
Mozolewski et al. [34]	2017	Indomethacin plus Isoflavonoid
Hauer et al. [35]	2007	Anesthetics, Tryciclic Antidepressants, Anticonvulsivants
Hauer et al. [36]	2010	Anesthetics, Tryciclic Antidepressants, Anticonvulsivants
Harrison et al. [37]	2017	Marijuana (Cannabinoids)

Rehabilitation management		
Source	Publication date	Rehabilitation therapies
Mishra et al. [48]	2017	Spinal Orthosis
Ravindran et al. [49]	2013	Complementary and Alternative medicine (CAM)
Vance et al. [50]	2014	Transcutaneous Electrical Nerve Stimulations (TENS)
Zhao et al. [51]	2008	Acupunture and Acupressure
Fisher et al. [52]	2018	Psychological Therapy
Winston et al. [53]	2005	Mindfullness, Excercises, Hypnosis, Educational programs, Biofeedback
Braun et al. [54]	2009	Animal-assisted therapy (AAT)
Kenny et al. [55]		Music therapy

Pharmacological/rehabilitation combined management		
Source	Publication date	Treatments
Congedi et al. [31]	2018	ERT, NSAIDs, Opioids, Cannabinoids, Physical/Psychological interventions

### Pain assessment in MPSs

Pain in children with MPS is assessed using different scales (Table 4). Fink, in 2000, presented the WILDA test (including Word to relate suffering, Intensity, Location, Duration, Aggravating elements), which can be used as an initial evaluation to put patients, above all children, at ease [18]. Politei et al., in 2018, suggested different scales that can be applied to MPS young patients, reporting which one to adopt based on the age of the subjects and their intellectual skills [19]. One of them is FLACC (Face, Legs, Activity, Cry and Consolability), a behavioural scale used for patients from two months to seven years of age and for those who have verbal difficulties in expressing their discomfort, such as neurologically impaired patients [20, 21].

**Table 4:** Pain assessment scales in MPSs according to patients’ age and intellectual development

Pain assessment scales in mpss		
Scales	Age	Further indications
FLACC [20, 21]	2 months–7 years	Behavioural scale adopted also for patients of any age neurologically impaired
FPS-R [22–24]	4 years–18 years	Self-evaluating numerical scales administered in collaborating patients without intellectual disabilities
NRS [22–24]	4 years–18 years	
CHAQ [22–24]	4 years–18 years	
NCCPC-R [16]	< 8 years	Scale used also for patients aged 3 to 18 years with mental and intellectual disabilities incapable to speak
WILDA [18]	> 8 years	Test used for initial pain assessment, putting patients at ease
BPI [25]	> 18 years	Inventory to distinguish moments of minimum ad maximum pain
SFHS-36 [26]	> 18 years	36 questions in 8 domains to evaluate pain impact on quality of life

For patients from four to eighteen years old, several scales are recommended: the Facial Pain Scale-Revised (FPS-R), the Numbering Rating Scale (NRS) and the Child Health Assessment Questionnaire (CHAQ). All these three scales adopt a self-evaluating numerical scale system of the pain severity experienced by the patient [22–24].

Brands et al. have shown the high reliability and validity of The Non-Communicating Children Pain Check List-Revised (NCCPC-R), a clinical instrument used to evaluate and measure pain in MPSs children aged < 8 years or aged 3 to 18 years, with mental and intellectual disabilities, incapable to speak. The NCCPC-R includes 30 items divided into seven subdomains: vocal, eating and sleeping, social, facial, activity, body and limb, physiological signs. Each item presents a numerical scale that parents or caregivers tick based on the frequency of pain in each situation [16].

For individuals older than 18, the Brief Pain Inventory (BPI) is suggested and includes different pain scores: the worst and the lightest pain suffered in the last 24 h, the average pain feeling over the past 24 h, the pain experienced by subjects at the moment of the assessment [25].

Finally, Ware and colleague proposes the Short Form Health Survey 36 questionnaire, which includes 36 questions in 8 domains and can be easily applied to investigate pain impact on quality of life [26].

### Musculoskeletal pain management in MPSs

#### Pharmacological therapy

##### Enzyme replacement therapy

Two trials reported the benefits of enzyme replacement therapy (ERT) on musculoskeletal pain management. In their 2009 randomized controlled trial, Clarke et al. tested the long term efficacy and safety of Laronidase in MPS I young patients (middle age 16 years). Good results in the Child Health Assessment Questionnaire (CHAQ) and in the Health Assessment Questionnaire Disability Index (HAQDI) were sustained over the treatment period, showing amelioration of joint mobility and decreased pain [27]. On the other hand, the 2015 randomised double-blind pilot study by Burton et al. showed good effects of two doses of elosulfase alfa in patients with Morquio A syndrome. The authors reported that the abnormal exercise capacity, the impaired muscle strength and the disabling pain observed at baseline tended clearly to improve after 27 weeks of treatment [28].

##### Pamidronate

In their 2003 case report, Robinson et al. proposed pamidronate, a biphosphonate which is commonly employed for the treatment of severe osteoporosis, to provide pain control in patients with MPS. Intravenous pamidronate treatment, given monthly for a year, produced dramatic clinical effects, with reduction in bone pain and improvement in mobility [29].

##### TNF inhibitor: adalimumab

In 2017, Polgreen et al. studied the effects of Adalimumab in subjects affected by MPS I and II in a 32-week, randomised, double blind, placebo-controlled, crossover study. Adalimumab was administered subcutaneously, every week for 16 weeks. The primary aim of the study was drug safety, while the secondary was the efficacy on reducing bodily pain (BP) measured by the the Children's Health Questionnaire—Parent Form 50 (CHQ-PF50). Two patients, one with MPS I and one with MPS II, completed the study and Adalimumab resulted well tolerated by them. Standardized BP scores for age and gender were higher (i.e. less pain) at the end of the treatment versus placebo for both subjects. Despite the low number of samples, Authors concluded that Adalimumab was safe, tolerable and may ameliorate pain in paediatric population affected by MPS I and II [30].

##### Analgesic drugs

According to World Health Organization (WHO), analgesic drugs for MPS patients include nonsteroidal anti-inflammatory drugs (NSAIDs), Acetaminophen and opioids (Table 5) [31]. Administration of these drugs depends on duration, worsening of pain and it is different if the pain suffered is neuropathic or nociceptive [32, 33]. NSAIDs and Acetaminophen are generally used first, alone in case of mild pain and associated with opioids in case of severe pain management. Among all NSAIDs, Ibuprofen and Ketoprofen are used for mild and occasional pain, while Diclofenac, Piroxicam, Naproxene and Indomethacin are used for moderate pain. As regards Indomethacin, Mozolewski et al. in 2017 reported that this drug, in association with an isoflavanoid compound, was capable to inhibit drastically GAGs synthesis in fibroblasts, influencing PI3K signalling pathways [34]. Finally, Ketorolac is the most effective NSAID for the management of severe pain in children with MPS. However, it could not be administered for long periods because of its side effects on gastric and medullar sites. Actually, Ibuprofen is the most frequently administered NSAID in MPS children because of its good safety profile [31]. Acetaminophen belongs to the “over-the-counter-analgesics” class and it is used for treatment of mild pain without causing important adverse effects.

**Table 5:** Drugs used and their posology [19]

Analgesics	DOSAGE
Acetaminophen	po: 20 mg/kg initially, then 15 mg/kg every 4-6 h rectal: 30–40 mg/kg initially, then 15–20 mg/kg every 4-6 h ev: weight < 10 kg: 7.5 mg/kg every 6 h weight > 10 kg: 15 mg/kg every 6 h Maximum dose: 90 mg/kg/day (60 mg/kg/day if present risk factors)
*NSAIDS*	
Low Power	
Ibuprofen	po: < 6 months: 5 mg/kg every 6-8 h 6 months: 10 mg/kg every 6-8 h rectal: weight > 6 kg, 60 mg suppository every 8 h weight > 12 kg, 125 mg suppository every 8 h Maximum dose: 40 mg/kg/day
Ketoprofen	po, rectal or ev: 3 mg/kg every 8-12 h Maximum dose: 9 mg/kg/day
Moderate Power	
Naproxene	po: 5–10 mg/kg every 8-12 h Maximum dose: 20 mg/kg/day
High Power	
Ketorolac	po: 0.2 mg/kg (max 10 mg) every 4-6 h ev, im: 0.5 mg/kg start, then 0.2–0.3 mg/kg every 4-6 h Maximum dose: 3 mg/kg/day
Indometacin	po, ev: 1 mg/kg every 8 h Maximum dose: 3 mg/kg/day
*OPIOIDS*	
Weak Opioids	
Codeine	po, rectal: 0.5–1 mg/kg every 4–6-8 h ATTENTION: NO if < 12y-old NO for 12–18 y-old if: Recent tonsillectomy and or adenoidectomy; Ultra-rapid metabolizer CYP2D6; Bad respiratory function
Tramadol	po: 0.5–1 mg/kg every 4–6-8 h ev: 1 mg/kg every 3-4 h ev: continuous infusion 0.3 mg/kg/h
Strong Opioids	
Morphine	CLORIDRATE (ev): Bolus 0.05–0.1 mg/kg every 2-4 h, Continuous infusion 0.02–0.03 mg/kg/h SOLFATE (po): Early release: 0.15–0.3 mg/kg every 4 h; Slow release: 0.3–0.6 mg/kg every 8-12 h
Oxicodone	po: 0.1–0.2 mg/kg every 8-12 h
Fentanyl	ev: Bolus 1–2 mcg/kg (max 5 mcg/kg with spontaneous breathing), Continuous infusion 0.1 mcg/kg/h Intranasal: 1–2 mcg/kg

In case of chronic moderate/severe pain involving adults, the pharmacological management includes opioids as Tramadol, Methadone, Oxycodone and adjuvant analgesics like the anticonvulsants Pregabalin and Gabapentin [31]. Nevertheless, their use in paediatric subjects is actually off-label [1]. In patients affected by severe incoercible pain, the use of pure mu opioid agonist is suggested: Morphine for episodic pain and Oxicodone, Fentanyl and Methadone for permanent severe pain [29, 31]. Neuropathic pain is difficult to manage: in this case, infusions of Ketamine and Lidocaine, antidepressants tryciclic as Amytriptyline, anticonvulsivants like Carmazepine and, again, Gabapentin are strongly suggested. In 2017 Hauer et al. showed the results of Gabapentin administration in 9 severely neurologically impaired young patients with a significant amelioration of hyperalgesia lasting from 3 months to 3 years [35, 36].

##### Cannabinoids

The 2013 review by Harrison et al. stated that there is a paucity of original research data regarding risks and benefits of marijuana use for treating chronic pain in adolescents. Although benefits may accrue in specific conditions, adverse effects influencing daily functioning often limit treatment [37]. Nevertheless, cannabinoids, especially phytocannabinoids, are increasingly a valid therapeutic option in the treatment of chronic pain. Braijyeh et al. reported that trans-∆-9-tetrahydrocannabinol (THC) and cannabidiol (CBD) are the most common cannabinoids present in the cannabis plant. In the body, phytocannabinoids bind to specific endocannabinoid receptors. Two of them, CB1 and CBD2 are the most distributed through human body: CBD1 mainly in immune tissues, brain and muscle; CB2 in skin and bones [38].

Nowadays, cannabinoids are being largely studied for central, nociceptive and neuropathic pain. CB1 receptors are abundant in nociceptive and non-nociceptive sensory neurons of the brain, the dorsal root ganglion (DRG) and the spinal cord. CB2 receptors are more represented in case of peripheral nerve lesion. Cannabinoids control pain through different mechanisms and receptors: THC is capable to prevent prostaglandin E-2 synthesis and induce lipoxygenase, riduce 5-hydroxytryptamine (5-HT) synaptosomal uptake and favourite its cerebral production [39], change dopaminergic function and activate vanilloid-transient receptor potential-2 (TRPV2). CBD can hamper the hepatic metabolism of THC to 11-hydroxy-THC, a compound more psychoactive, increasing its half-life and decreasing its side effects [40]. CBD is an endocannabinoid modulator and gets anti-inflammatory effects by reducing reactive oxygen species (ROS), TNF-α levels and pro-inflammatory cytokines; preventing T cell proliferation, migration and adhesion of immune cells; easing T cell apoptosis [38, 41]. CB1 and CB2 receptors provide for the inflammatory suppression CBD-mediated. CBD, however, has low affinity for CB1 and CB2 but enhances anandamide, an endogenous cannabinoid with higher affinity for CB1 and CB2 receptors. Furthermore, CBD gets immune effects mediated through a blockade of GPR55 receptors [42]. The G protein-coupled receptor 55 (GPR55), is a lysophosphatidylinositol (LPI)-sensitive receptor involving in cannabinoid signaling. It is present, above all, in the periaqueductal gray (PAG), a brainstem area necessary for the descending pain control system [43]. GPR55 is a seven transmembrane-spanning domain which activates a G-protein Gαq/12 and Gα13. GPR55 can stimulate different transcriptional networks, also those mediated by CREB and NFkB [44, 45]. GPR55 is widely represented in the central nervous system (CNS) and plays an important role in central physiology and pathology. A potential involvement of GPR55 in pain has been shown mainly through a knockout mice approach [46]. Armin et al., in 2021, employed antagonists to explore the role of GPR55. Their data reported that, in the neuropathic pain model in rats, it is doable to induce the descending control system to decrease neuropathic pain through the pharmacological blockade of the function of GPR55 in the PAG. They showed that in the above model GPR55 has a primary role in the descending pain control: by blocking the GPR55 action in the PAG, in fact, an inhibition of pain by the descending pain control system, rather a facilitation of the same, is achievable. Further, these findings disclose that the descending pain control system is yet available for pain control because, in the PAG, GPR55 action’s blockade achieves analgesic effects [47]. It is desirable to apply these findings on cannabinoids in order to develop novel and improved therapeutic strategies for alleviating chronic neuropathic pain, not only in older but also in paediatric patients.

#### Rehabilitation management

A case report, conducted by Mishra et al., reported good outcomes on the treatment of kyphotic deformity. They tested, in a 2-year-old subject affected by Hurler syndrome, a spinal orthosis made of ethaflex and with polypropylene able to reduce a prominent gibbus from 50° to 20°, hold up paravertebral muscles and let the little patient regain autonomy in standing and walking [48].

From the article review of Congedi et al. in 2018 concerning drug therapies and rehabilitation techniques, physical therapy—such as heat/cold applications, osteopathy, physiotherapy, massage and chiropractic—have showed satisfactory outcomes in acute and chronic pain control. It is able to reduce suffering also in paediatric population and to increase their self-confidence [31]. Ravindran et al. in 2018, instead, in their systematic review have summarised the effects of complementary and alternative medicine (CAM) on depression, panic and anxiety disorders [49].

Vance et al. in their review have observed a better control of hyperalgesia and allodynia [50] in patients who received transcutaneous electrical nerve stimulations (TENS), which is a non-pharmacological approach stimulating a complex neuronal rate to inhibit the descending ways of the central system and thus to decrease pain.

In the review of Zhao et al. in 2008, acupuncture and acupressure have also been proposed in this population. These treatments are based on acupoint stimulation that results in analgesia inhibiting release of pain peptides from nucleus raphe magnus [51].

Winston et al. in their review have analysed psychological therapies such as hypnosis, biofeedback, desensitisation, stress management, relaxation, mindfulness exercises and educational programs, which can be carried out alone or in group and proceed to decrease chronic pain and to face up suffering activities [52, 53]. Fisher et al., instead, in a review, have assessed the psychological therapies effects on chronic and recurrent pain in children with MPS experiencing mixed pain, which resulted in a decrease of pain perception post-treatment, but these effects were not maintained at follow-up. Moreover, they reported good results on reducing disability for young people with mixed pain conditions post-treatment and at follow-up [52].

The clinical trial by Braun et al. concerning animal assisted therapy (AAT), as well as the clinical trial of Kenny et al. concerning music therapy, suggest alternative rehabilitative approaches with several positive effects [54, 55]. The AAT assisted therapy, indeed as observed by Braun et al., reduced pain intensity and improved respiratory parameters notably in children between 3–17 years, in one acute care paediatric setting [54].

## Discussion

Patient with mucopolysaccharidoses, besides its seven types, experiences chronic pain starting since childhood and sometimes it can even reach very high intensities [16]. In clinical practice it is important to assess the visceral and musculoskeletal pain to adopt the best therapeutic approach to control or reduce it for each level. That is why in literature are always more often published articles concerning the management of rare conditions, which has also the purpose of increasing quality of life [56, 57]. Investigating and analysing MPS pain is not very simple because each patient has a different grade of intelligence and a wide spectrum of phenotypes [58], especially in children. The test type more frequently used, especially in first instance, adopts a self-evaluating numerical scale that can be compiled by the patient himself (i.e. BPI, FPS-R) [22–25] or by a parent/caregiver in peculiar situations—for example, when the patient can’t speak (i.e. NCCPC-R) [16]. When, instead, the patient is too young and so not self-conscious, the best way to assess pain is adopting a behavioural test (i.e. FLACC) [20, 21]. Questionnaires, on the other hand, are usually adopted in order to assess the impact of pain on quality of life [26].

As far as the pharmaceutical approach, for MPS I, II, IV and VI, the ERT – like Laronidase and elosulfase alfa—represents one of the most used approach to control pain worldwide, indeed, in the studies examined patients referred a good pain control from this [28, 58]. However, according to some researchers, when the developmental quotient is high, a hematopoietic stem cell transplantation (HSCT) should be preferred [59]. In addition, the HSCT therapy has shown improvement particularly in regard to neurologic deterioration [60, 61]. The bisphosphonate Pamidronate, according to the literature, is commonly used to reduce bone pain and improve mobility [29]. Another class of drugs frequently used are the anti-inflammatory because inflammation represent a hallmark of MPS etiopathogenesis and high levels of TNF-α are certainly related to reduced motor ability and raised joint pain [62]. Adalimumab, indeed, could be a good alternative for pain MPS management because it is safe and tolerable [30]. NSAIDs are recommended when pain is mild, but not for long periods due to their adverse effects (e.g. Ketorolac), except for Ibuprofen and Acetaminophen which are considered safer [31–34]. Indomethacin combined with an isoflavanoid compound has little evidence to inhibit GAGs synthesis [34] but more studies are needed to confirm this hypothesis. When instead pain reaches a severe intensity, transmucosal and intravenous opioids – like Tramadol, Methadone, Oxycodone, Pregabalin, Gabapentin, Morphine and Fentanyl – are recommended to successfully decrease pain, acting directly on Central Nervous System (CNS) [4, 29, 31, 35, 36]. Cannabinoids also might have a role in pain control in young adults [37], yet their administration in paediatric patients is still controversial due to the adverse psychophysical reactions like pain attacks, paranoia and neuromotor impairment. Nevertheless, a study has observed instead how marijuana could actually be a solution to the psychological problems that adolescent with diseases are forced to face [63]. Neuropathic pain remains difficult to manage in this population, but among all opioid drugs the Gabapentin is the one with the highest evidences in literature concerning the amelioration of hyperlagesia [35, 36].

However, while drug therapies listed above have only a palliative purpose, rehabilitation could provide cope strategies helping the patients to manage their pain by themselves. In literature, different techniques have been experimented for pain management in MPS younger patients. Physical therapy, which includes yoga, physical exercise, osteopathy, heat and cold application and massage, is one of the most versatile and important rehabilitation approaches, capable of effecting pain control, increasing self-confidence and containing psychological disorders like anxiety and depression [31, 39]. It is very important to train the patient in order to help him with ambulation and postural alignment, but also to prevent the formation of contractures and the progression of scoliosis [17]. In case the disease causes a kyphotic deformity, a spine anomaly very painful and disabling for children owing to aesthetic, kinaesthetic and respiratory aspects, a spinal orthosis made of ethaflex and polypropylene could be a supportive treatment when cervical spine compression is not at high levels [48]. TENS may also play an effective role in controlling hyperalgesia and allodynia [50]. Acupuncture and acupressure have been proposed too, because they induce analgesia inhibiting the release of pain [51]. However, there are no enough evidences to support this treatment. Since MPS may lead to neurocognitive impairments such as behavioral abnormalities, sleep problem and/or seizures [64], psychological integrated approaches, which involves educational and behavioural programs, mindfulness exercises and relaxation, may also be a relevant strategy to contain the stress level of these patients and to prevent the aggravation of the symptoms. They can also be extended to families in order to support them and lighten the burden of the disease [65, 66]. Notwithstanding the benefits showed, therapeutic use of these approaches is limited by their excessive cost and by socio-cultural elements concerning patients and their families/caregivers [52, 53]. Finally, the animal assisted therapy and music therapy have light evidences supporting their role in pain management of children in acute phase [54]. These last two therapies have a very fascinating role and are very promising for children for many reasons. First, because they do not always require a therapeutic setting and so they can be practiced anywhere and anytime. Second, because they are very enjoyable for children, and so it is hard for them to refuse or reject them. At last, they could provide long terms effects and implement strategies without any adverse effects.

### Limitations

Due to the shortage of data in literature, this review cannot make clinical recommendations regarding specific pharmacological and rehabilitation interventions on the topic examined. Where by, the findings of this review should be interpreted with caution due to the small sample size across the studies. Additionally, the previous mentioned inclusion criteria are limited to peer-reviewed journals written in the English language, thereby excluding significant work that could have been published in other languages. However, the above data may encourage the conduction of a greater number of studies in order to increase the reliability of the data reported by this scoping review.

## Conclusion

In conclusion, pain afflicts a great many paediatric MPS patients but, it is still little, badly analysed and undertreated. Validated age specific rating scales are needed to classify pain intensity in order to formulate the appropriate treatment for each level. Different pharmacological therapies are available to control musculoskeletal pain, but the most effective are the ERT, NSAIDs and Acetaminophen for mild pain, while oral, transmucosal and intravenous opioids for the severe one. Cannabinoids are still controversial as they control successfully pain refractory to other drugs, but have notable psychophysical adverse effects in paediatric age. Increasing importance should be reserved, in the future, to the multidisciplinary approach of rehabilitation (i.e., physical, psychological and music therapies) which are of great support in paediatric population, since they are applicable in the long term without side effects compared to drugs, becoming integral part of lifestyle of MPS children. High-quality studies are warranted to refine the uncertain estimates in this review.

## Data Availability

The data that support the findings of this study are available from https://pubmed.ncbi.nlm.nih.gov/ but restrictions apply to the availability of these data, which were used under license for the current study, and so are not publicly available.
